# Seasonal Variation of the Proximate Composition, Mineral Content, Fatty Acid Profiles and Other Phytochemical Constituents of Selected Brown Macroalgae

**DOI:** 10.3390/md19040204

**Published:** 2021-04-04

**Authors:** Marco Garcia-Vaquero, Gaurav Rajauria, Marta Miranda, Torres Sweeney, Marta Lopez-Alonso, John O’Doherty

**Affiliations:** 1 School of Agriculture and Food Science, University College Dublin, Belfield, Dublin 4, Ireland; marco.garciavaquero@ucd.ie (M.G.-V.); gaurav.rajauria@ucd.ie (G.R.); 2 Department of Anatomy, Animal Production and Clinical Veterinary Sciences, Faculty of Veterinary, Universidade de Santiago de Compostela, Campus Terra, 27002 Lugo, Spain; marta.miranda@usc.es; 3 School of Veterinary Medicine, Veterinary Science Centre, University College Dublin, Belfield, Dublin 4, Ireland; torres.sweeney@ucd.ie; 4 Department of Animal Pathology, Faculty of Veterinary, Universidade de Santiago de Compostela, Campus Terra, 27002 Lugo, Spain; marta.lopez.alonso@usc.es

**Keywords:** seaweed, fatty acid, mineral, antioxidant, climate, proximate analysis, nutrient profiling

## Abstract

The main objective was to determine the chemical, phytochemical, fatty acid and mineral profiles of three commercially relevant brown macroalgae (*Laminaria digitata*, *Laminaria hyperborea* and *Ascophyllum nodosum*) collected each season for two years off the west coast of Ireland. All the chemical, phytochemical, fatty acid and minerals analysed varied significantly depending on the macroalgal species, season and year of collection. Overall, the protein contents of macroalgae were negatively correlated with carbohydrate content. Protein (2–11%) was at its highest during winter and/or spring, decreasing to a minimum during summer and/or autumn. The three macroalgal species analysed in this study had clearly differentiated fatty acid profiles. The concentration of fatty acids was higher in *A. nodosum* compared with both Laminaria species. The mineral profile of the three macroalgal species was rich in essential metals, particularly Ca, Mg and P, while the levels of I were approximately 9- to 10-fold higher in both *Laminaria* spp. compared with *A. nodosum*. The levels of toxic metals (Cd, Hg and Pb) in all the macroalgal species studied were low in the current study; while the levels of total As were high (49–64 mg/kg DW macroalgae) compared with previous reports.

## 1. Introduction

Over 10,000 species of macroalgae have been identified worldwide; however, only 5% of these are currently exploited for food or animal feed applications [1]. Overall, the scientific literature describes the macroalgal biomass as rich in carbohydrates (up to 60%), with medium or high amounts of proteins (10–47%), low in lipids (1–3%) and variable contents of minerals (7–38%) [2]. Macroalgae are able to adapt to the rapid changes of the marine environmental conditions, such as changes in temperature and solar radiation by producing unique secondary metabolites, including polysaccharides, proteins, lipids and phenolic compounds [3]. Prolonged exposure of macroalgae to environmental stressors, such as fluctuations in water level, solar radiation and temperature, can lead to the formation of reactive oxygen species and other free radicals in the biomass. As a defence mechanism, the stressed macroalgal biomass produces high amounts of antioxidant compounds, such as phenolic compounds and sulphated polysaccharides amongst others, trying to maintain the integrity of the cellular structures [4]. Thus, the macroalgal biomass is rich in a wide variety of antioxidant compounds which can be incorporated in human nutrition as food or supplements. They can also provide additional health benefits to those of basic nutrition—namely, nutraceuticals or functional foods [5] that could help in the prevention of chronic diseases, such as cancer, cardiovascular diseases, obesity and diabetes [6,7].

Macroalgal carbohydrates and lipids include molecules of diverse chemical nature and biological roles in the biomass. Macroalgal polysaccharides are a diverse group encompassing dietary fibres and other soluble carbohydrates, such as glucans and fucoidan, with promising health benefits, including anti-inflammatory, antioxidant and antitumor properties in vitro and in vivo [8]. Amongst lipids, macroalgae are considered a rich source of polyunsaturated fatty acids (PUFA), such as omega-3 and 6 fatty acids, which have been linked with promising health benefits, including improvement of maternal and offspring health, growth and development, cognitive function and psychological status [9,10]. Macroalgae have also been reported to accumulate a high amount of metals, including essential metals (calcium (Ca), cobalt (Co), chromium (Cr), copper (Cu), iron (Fe), iodine (I), magnesium (Mg), manganese (Mn), molybdenum (Mo), nickel (Ni), phosphorus (P), selenium (Se) and zinc (Zn)), with relevant properties in maintaining human health when included at appropriate levels in the diet. However, macroalgae are also known to accumulate toxic metals, such as arsenic (As), cadmium (Cd), lead (Pb) and mercury (Hg), which are highly toxic even at trace levels and rank among the priority metals with public health significance [11,12,13,14,15,16].

The composition of macroalgae is extremely variable depending on the macroalgal class (i.e., green, red, or brown macroalgae) [17,18], species [19,20], stage of development of the biomass (i.e., sterile versus fertile tissue) [21] and environmental stressors, such as the season of collection [4], which will influence the biology, and thus the composition of macroalgae. The elucidation of these changes in composition could be especially relevant for studies targeting the extraction of high-value compounds from this biomass for nutraceutical applications.

The main objective of the present study was to determine the chemical, phytochemical, fatty acid and mineral profiles of three commercially relevant brown macroalgae (*Laminaria digitata*, *Laminaria hyperborea* and *Ascophyllum nodosum*) collected each season off the west coast of Ireland, aiming to gain an insight into the key environmental factors affecting the macroalgal wild biomass in Ireland.

## 2. Results and Discussion

### 2.1. Proximate Composition, Phytochemical Concentration and Antioxidant Capacity

The proximate composition (dry matter (DM), ash, gross energy (GE), protein, total soluble sugars (TSS), neutral detergent fibre (NDF), acid detergent fibre (ADF) and ether extract (EE)) of dried and milled *L. digitata*, *L. hyperborea* and *A. nodosum* collected each season during the years 2016 and 2017 is presented in Table 1. All of the parameters analysed varied widely depending on the seaweed species, season and year of collection. Overall, the ash contents of the samples ranged from 17.9% to 36%, being in general more consistent through the seasons in *A. nodosum* compared with the other two Laminaria species. In general, the macroalgae of this study had the highest levels of ash during spring, except in the case of *L. digitata* collected in 2016, and all the *A. nodosum* samples had the highest levels of ash during winter. Previous literature reviews also described levels of ash in brown macroalgae varying between 15% and 45% [22]. Moreover, Bikker et al. [23] reported ash contents ranging from 18.9% to 37.4% in samples of *L. digitata* and *A. nodosum* collected in Ireland and France. The high ash contents in macroalgae will hamper the inclusion of intact seaweed in human and animal diets at high inclusion levels and may indicate the need to extract polysaccharides, protein and other relevant nutrients to decrease the levels of minerals added to the diet [23]. A complete mineral profile of the seaweed samples is described later in Section 2.3. The high levels of ash together with low level of lipids underpin the low energy value (GE 10.78 to 15.99 MJ/kg DW macroalgae) of the biomass analysed in this study. This is similar to previous reports in macroalgae [24]. The EE of the macroalgal samples in this study ranged from <0.1% to 3.82%, being maximum always in *A. nodosum*, with lipid contents of about 10-fold and 4-fold to those described in *L. digitata* and *L. hyperborea*, respectively. Further fatty acid analyses were performed, and nutritionally relevant fatty acids and lipid fractions from macroalgae are presented in Section 2.2.

Fibre is an important constituent of a number of food ingredients, and it was measured in this study by determining NDF and ADF contents. However, the detergent methods do not fully recover soluble fibres, and thus the actual fibre content is therefore underestimated [25]. The levels of fibre in the macroalgal samples of this study ranged from 27% to 67% for NDF and 11% to 35% for ADF with variable levels depending on the macroalgal species, season and year of collection and no clear pattern to allow us to draw conclusions on fibre content. Overall, the levels of both NDF and ADF were similar to those described in other brown macroalgae [24]. The levels of NDF were in all cases higher than ADF, similar to terrestrial plants.

**Figure 1:**
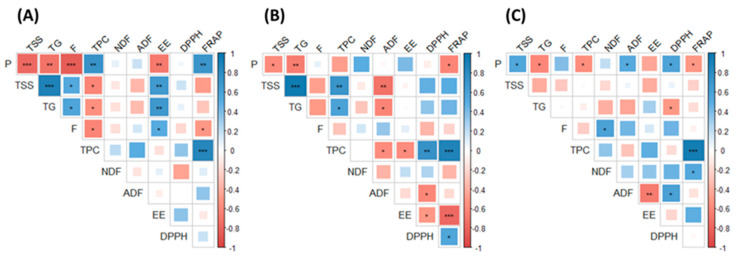
Correlation matrices of the main composition and antioxidant parameters analysed in (**A**) *L. digitata*, (**B**) *L. hyperborea* and (**C**) *A. nodosum*. The positive correlations are indicated in blue, while the negative correlations are indicated in red. The size of each square and depth of each colour indicate the strength of the correlations (0–1). Abbreviations in the figure are as follows: NDF (neutral detergent fibre), ADF (acid detergent fibre), P (protein), EE (ether extract), F (fucoidan), TSS (total soluble sugars), TG (total glucans), TPC (total phenolic content) and antioxidant properties (DPPH and ferric reducing antioxidant power (FRAP)). The statistical significance of the correlations is indicated in the figure as * *p* < 0.05, ** *p* < 0.01, *** *p* < 0.001.

The protein contents (ranging from approximately 2% to 11%) and TSS (≈11% to 27%) of the macroalgal samples of this study were also in agreement with previous reports [22]. In general, the protein levels of the macroalgal species in this study were lower than those described in other protein rich crops (i.e., pulses 21–25%) [26], and thus the use of the macroalgae collected in this study as a protein rich ingredient to enrich food formulations with this macronutrient may be limited. The accumulation of both protein and TSS varied depending on the macroalgal species; however, overall, the protein content in all macroalgae species in this study was at its highest during winter and/or spring, decreasing to a minimum during summer and/or autumn, while the accumulation of TSS followed an opposite accumulation pattern to that described for proteins. Moreover, the concentration of protein was negatively correlated with TSS and/or total glucans (TG) in the three macroalgal species studied (see Figure 1). A similar accumulation pattern of carbohydrates and proteins was previously described by Schiener et al. [27] when exploring the accumulation of multiple soluble carbohydrates and protein in *L. digitata* and *L. hyperborea*.

**Figure 2:**
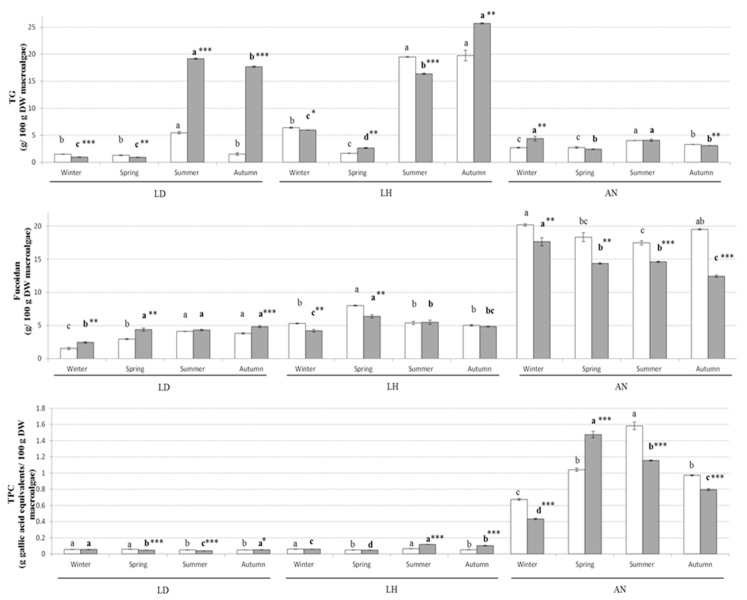
Phytochemical composition (TG, fucoidan and TPC) of *L. digitata* (LD), *L. hyperborea* (LH) and *A. nodosum* (AN) collected each season during the years 2016 (white bars) and 2017 (grey bars). Results are expressed as average ± standard deviation of the mean. Different letters indicate statistical differences (*p* < 0.05) in the phytochemical contents of each macroalgae between different seasons within the same year 2016 (regular letters) or 2017 (bold letters). The differences in phytochemical composition within the same season between the years 2016 and 2017 are indicated as follows: * *p* < 0.05, ** *p* < 0.01 and *** *p* < 0.001.

**Figure 3:**
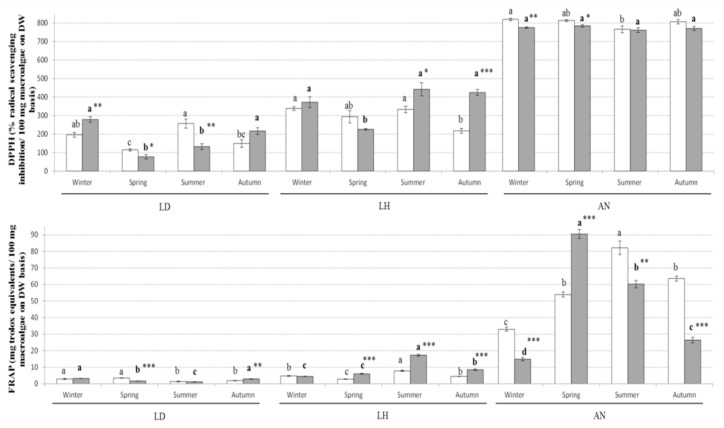
Antioxidant properties (DPPH and FRAP) of *L. digitata* (LD), *L. hyperborea* (LH) and *A. nodosum* (AN) collected each season during the years 2016 (white bars) and 2017 (grey bars). Results are expressed as average ± standard deviation of the mean. Different letters indicate statistical differences (*p* < 0.05) in the antioxidant activities of each macroalgae between different seasons within the same year 2016 (regular letters) or 2017 (bold letters). The differences in antioxidant activity within the same season between the years 2016 and 2017 are indicated as follows: * *p* < 0.05, ** *p* < 0.01 and *** *p* < 0.001.

Similar to the proximate composition parameters, there was significant variation in the accumulation of TG, fucoidan, total phenolic content (TPC) and antioxidant activities (DPPH and ferric reducing antioxidant power (FRAP)) depending on the macroalgal species, season and year of collection (Figure 2 and Figure 3). In general, the amount of total glucans was higher in *L. digitata* and *L. hyperborea* compared with *A. nodosum*, with high levels during the summer and autumn and low levels in winter and spring. This is similar to the previous seasonal changes described in multiple soluble carbohydrates in macroalgae by Schiener et al. [27]. The authors described a high accumulation of laminarin during the summer and autumn months (reaching a maximum of 25% in *L. hyperborea*) that dropped to its lowest levels (1–3%) during the winter, while the accumulation of protein had an opposite behaviour in both *L. digitata* and *L. hyperborea* [27].

In the case of fucoidan, TPC and antioxidant activities, *A. nodosum* had in general higher levels, up to 4-fold more, compared with the two Laminaria species. The levels of fucoidan in *A. nodosum* were in general high during winter, with variable concentrations appreciated depending on the year of collection. Previous studies investigating the seasonal variation of fucoidan in *Fucus serratus*, *Fucus vesiculosus* and *A. nodosum* reported high levels of fucoidan during autumn, with low concentration of this compound during spring [28]. A high proportion of sulphate in fucoidan molecules that may influence the biological properties of these molecules has also been reported when extracting these compounds during the winter season compared with the summer [28]. Moreover, the molecular weight of fucoidan as determined by Fletcher et al. [28] were fairly constant over the year in brown macroalgae (*F. serratus*, *Fucus vesiculosus* and *A. nodosum*). Thus, when harvesting and exploiting brown macroalgae as a source of fucoidan, important consideration should be paid to the season of collection. The levels of TPC and FRAP antioxidant activity in *A. nodosum* in the current study were in general low during the winter, increasing during spring and summer and declining during autumn, while no particular trend was appreciated in the case of DPPH. A prolonged exposure of macroalgae to environmental stressors can lead to the formation of reactive oxygen species and other free radicals in the biomass [4]. Thus, the high levels of TPC and antioxidant activities produced by the macroalgal biomass during spring and summer can be related to a defence mechanism of macroalgae against oxidative damage, helping to maintain the integrity of the cellular structures. Moreover, the concentration of TPC and the antioxidant properties (FRAP and/or DPPH) in this study were strongly and positively correlated in the three macroalgal species studied (see Figure 1). These results were in agreement with the seasonal variation appreciated in *A. nodosum* collected monthly during 2005 on the coast of Scotland [29]. The authors attributed the variation in TPC to the different phases of growth and reproductive stage of *A. nodosum*, with high levels produced during April and June as the biomass reaches the fertility stage [29].

**Figure 4:**
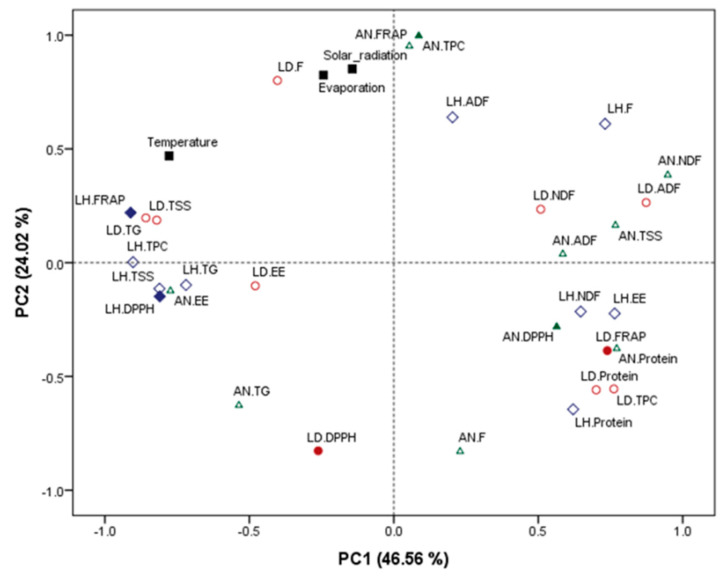
Principal component analysis scatter plot representing the scores for the climatological data (temperature, solar radiation and evaporation) in macroalgae (LD (*L. digitata*, red colour), LH (*L. hyperborea*, purple colour) and AN (*A. nodosum*, green colour)) and their proximate composition, phytochemical and antioxidant properties. Abbreviations in the figure are as follows: TSS (total soluble sugars), NDF (neutral detergent fibre), ADF (acid detergent fibre), F (fucoidan), TG (total glucans) and TPC (total phenolic content).

A principal component analysis (PCA) was performed to obtain an overview of the similarities and differences in the proximate, phytochemical and antioxidant composition of the three brown macroalgal species and the climatological data monitored in the region during those years (see Figure 4). Principal component 1 (PC1) explained 46.56% of the variation of the data set and PC2 explained 24.02%. The PC1 seems to separate multiple parameters between both Laminaria species, although no clear pattern or association can be made from the data set. The PC2 further separates the variation of the data set by clustering the climatological parameters, solar radiation and evaporation with the levels of TPC and FRAP antioxidant activity in *A. nodosum*. An increased solar irradiance and exposure to UV during the summer have also been linked to an increased production of antioxidant compounds in other intertidal macroalgal species [30,31]. Moreover, Pavia and Toth [32] concluded that exposure to sunlight had a positive effect on the content of phenolic compounds, such as phlorotannins, in natural and cultivated *A. nodosum* and *F. vesiculosus*.

### 2.2. Fatty Acid Profiling

Thirty-four fatty acids were identified and quantified in *L. digitata* (Supplementary Table S1 online), *L. hyperborea* (Supplementary Table S2 online) and *A. nodosum* (Supplementary Table S3 online) samples. There was significant variation in the individual concentration of fatty acids and in the overall accumulation of saturated fatty acids (SFA), monounsaturated fatty acids (MUFA) and polyunsaturated fatty acid (PUFA) in the samples depending on the macroalgal species, season and year of collection. In general, the concentration of SFA, MUFA and PUFA were higher in *A. nodosum* compared with the other 2 macroalgal species. *A. nodosum* had high levels of SFA ranging from 43.91 to 58.47 mg per kg DW macroalgae during the years 2016–2017, while the levels of SFA were low in *L. digitata* (23.98–32.28 mg/kg DW macroalgae) and *L. hyperborea* (19.54–30.49 mg/kg DW macroalgae) collected within the same timeframe. Similarly, the concentration ranges of MUFA (72.43–123.55 mg/kg DW macroalgae) and PUFA (40.79–56.34 mg/kg DW macroalgae) were higher in *A. nodosum* compared with the other two Laminaria species, with MUFA and PUFA levels ranging from 12.13 to 21.64 and 6.46 to 31.04 mg/kg DW macroalgae, respectively. These results are in agreement with previous literature reporting a wide variability in the accumulation of fatty acids in macroalgae depending on the macroalgal species and parts of the macroalgae sampled, together with variations reported due to environmental factors related to the season, collection site and nutrient availability [33,34,35,36].

Amongst the SFAs, the levels of C16:0 were high followed by C14:0 and C17:0 compared with the other SFA identified in *L. digitata* and *L. hyperborea*, while *A. nodosum* accumulated high levels of both C16:0 and C14:0, followed by C17:0. The most abundant MUFAs described in all the macroalgal samples collected in this study were oleic acid (C18:1, cis9; ω9), followed by palmitoleic acid (C16:1, cis9; ω7) with variable levels depending on the year and season of collection. These results were similar to previous reports on brown macroalgae [35,37].

Overall, the main fatty acids described amongst all the PUFAs quantified in this study included the omega-3 (ω3) fatty acids, α-linolenic acid (ALA; C18:3) and eicosapentaenoic acid (EPA; C20:5 cis5,8,11,14,17) and the ω6 linoleic acid (LA; C18:2, cis9,12) and arachidonic acid (ARA; C20:4). The PUFA profile varied significantly depending on the macroalgal species. In the case of *L. digitata*, the levels of these four fatty acids were all higher when compared with the remaining identified PUFA. While in *L. hyperborea*, ARA and EPA were higher than LA and ALA, followed by the remaining PUFA. In the case of *A. nodosum*, the amounts of ARA and LA were comparable through the seasons and higher than EPA and ALA. Dawczynski, Schubert and Jahreis [37] reported high accumulation of PUFA representing between 31.8% and 74.7% of the total fatty acids when analysing 34 species of red and brown macroalgae. The authors also emphasised the high levels of ω3 in macroalgae, with high levels of EPA in the brown macroalgae *Hizikia fusiforme* and other red macroalgal species [37]. Previous reports also suggest that macroalgae grown in cold climate countries could have better lipid composition compared with those grown in warm waters due to an increased accumulation of PUFA [36,38]. The relative abundance of PUFA in the macroalgae analysed in this study is relevant for human nutrition due to the promising health benefits of these compounds when included in the diet. Even if it remains questionable whether the incorporation of macroalgae in the diet could represent a significant contribution towards fulfilling the dietary requirements of ω3 [39], their inclusion into the diet can make a significant contribution towards achieving the recommended ω6/ω3 ratios. European nutritional societies recognize a ratio of 5:1 (*ω*6/*ω*3) in the diet as health-promoting, with most Western diets deficient in ω3 with ratios of about 15–17:1 [37,40,41]. Therefore, the ω6/ω3 ratios of *L. digitata* (0.77–1.28:1), *L. hyperborea* (0.67–0.86:1) and *A. nodosum* (2.35–3.75:1) analysed in the current study could be useful for improving PUFA dietary ratios and potentially reducing or preventing numerous chronic diseases [42,43,44].

**Figure 5:**
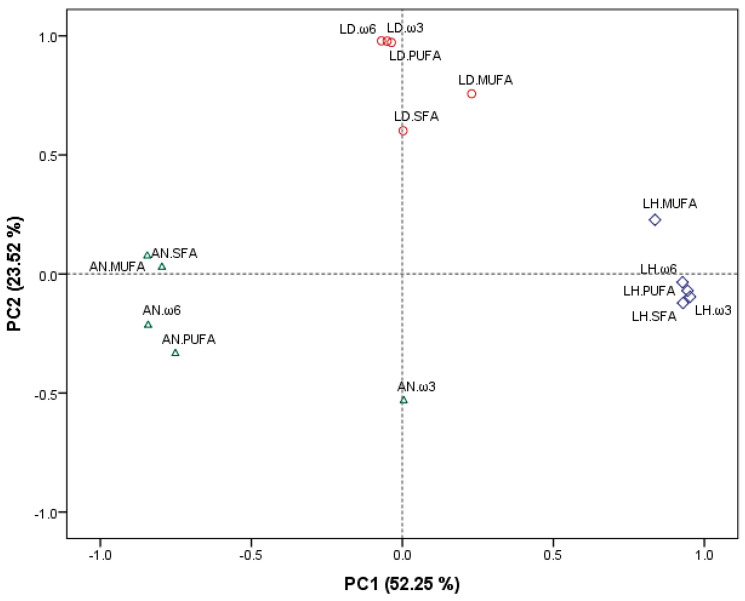
Principal component analysis scatter plot representing the scores for the fatty acid composition of macroalgae LD (*L. digitata*, red colour), LH (*L. hyperborea*, purple colour) and AN (*A. nodosum*, green colour). Abbreviations in the figure are as follows: SFA (saturated fatty acids), MUFA (monounsaturated fatty acids), PUFA (polyunsaturated fatty acids), ω3 (omega 3 fatty acids) and ω6 (omega 6 fatty acids).

Principal component analysis (PCA) was performed to obtain an overview of the similarities and differences in the fatty acid profiles of SFAs, MUFAs and PUFAs, including the total ω3 and ω6 of the macroalgal species studied. The two PCs obtained from the data explained 75.77% of the cumulative variation of the data set, being 52.25% and 23.52% explained by PC1 and PC2, respectively (see Figure 5). PC1 separates clearly the fatty acid profile of *L. hyperborea* from the other two macroalgal species, with all the values of *L. hyperborea* clustered on the right side of PC1, while the fatty acids of *A. nodosum* are grouped on the opposite side of PC1. The second component explained further the variability of the data set and separated further the fatty acid contents of *L. digitata* from those of the other two macroalgal species. Overall, the PCA results of this study indicate a clear differential distribution pattern of fatty acids depending on the macroalgal species. Previous studies identified differential accumulation patterns of ω3 and ω6 at phylum, order and family taxonomic levels in macroalgae [17]. The results of the current study could indicate the potential of the fatty acids profiles of macroalgae, particularly the ω3 and ω6 that cannot be synthesised by animals [45,46], to be used as biomarkers of macroalgal consumption.

### 2.3. Essential and Toxic Trace Metals

The levels of essential (Ca, Co, Cr, Cu, Fe, I, Mg, Mn, Mo, Ni, P, Se and Zn) and toxic (As, Cd, Hg, Pb and Sr) trace metals monitored in *L. digitata* (Supplementary Table S4 online), *L. hyperborea* (Supplementary Table S5 online) and *A. nodosum* (Supplementary Table S6 online) collected in this study varied significantly depending on the macroalgal species, season and year of collection. Similar findings and variable levels depending on the season were also reported by Khaled et al. [47] and Roleda et al. [4].

Amongst the essential metals, the levels of Ca, Mg and P were high and comparable in all macroalgae, while the levels of I were approximately 9- to 10-fold higher in the two *Laminaria* spp. compared with *A. nodosum*. Similar findings with the high content of I in *Laminaria* spp. were previously reported [19,48]. Biancarosa et al. [19] reported levels of I ranging from <200 mg/kg DW in most red algal species to >3000 mg/kg DW in some brown algal species, with the levels of *L. digitata* being the highest within brown macroalgae, reaching 10,000 mg/kg DW, similar to those reported in the current study. The levels of I in both *Laminaria* spp. in the current study showed high accumulation of this metal in the winter with decreased levels in the summer–autumn, a similar pattern to that described in previous studies [49,50]. Moreover, previous reports also established a correlation between the decrease of the antioxidant defences of macroalgae provided by iodine during the summer, with the need of macroalgae to synthesize and accumulate a high amount of other antioxidant molecules, such as TPC, to protect the biomass from the increased sun irradiation and oxidative species [29,30,31].

The contents of other essential metals were in agreement with previous reports analysing the mineral contents of macroalgae [19]. The levels of Fe ranging from 46 to 238 mg/kg DW macroalgae and Zn from 22 to 78 mg/kg DW macroalgae were comparable between the three macroalgal species. Other essential trace elements analysed (Co, Cr, Cu, Mn, Mo, Ni and Se) were low, and in the case of Mo, approximately 37% of the readings were below the detection limits of the method.

The macroalgae of this study accumulated low levels of toxic metals, Cd (0.07–1.63 mg/kg DW macroalgae), Hg (0.01–0.06 mg/kg DW macroalgae) and Pb (0.01–1.11 mg/kg DW macroalgae), with no clear differences amongst the three macroalgal species studied. All the samples analysed in this study were below the maximum admissible levels for Cd established by the European Commission for food supplements, consisting mainly of dried macroalgae or products derived from this biomass of 3 mg/kg [14]. In the case of Hg and Pb, no specific limits have been set for macroalgae in food. The levels of Hg analysed in this study were all below the maximum admissible levels established for fishery products (0.5 mg/kg), while the levels of Pb were above those established for vegetables (0.1 mg/kg) and other marine products, such as molluscs (1.5 mg/kg) [15].

The levels of total As in the samples of this study were within the higher accumulation ranges when compared with previous scientific literature [19,51]. The accumulation of As was higher in the two Laminaria species, ranging from 75 to 615 mg/kg DW macroalgae, compared with *A. nodosum* (49–64 mg/kg DW macroalgae). Previous reports emphasized the role of fish, seafood and macroalgae as the major worldwide food sources of total arsenic [12]. Currently, no maximum levels have been set for the levels of As in foodstuff in Europe [15]. However, in the case of macroalgae being used as a feed material, the maximum levels of total arsenic allowed are 40 mg As per kg of feedstuff with a moisture content of 12%, demonstrating upon request by the authorities that the content of inorganic arsenic is lower than 2 mg/kg in feed, particularly when using the brown macroalga *Hizikia fusiforme* [52]. Thus, the levels of As may indicate the need to perform future As speciation studies to quantify the amount of inorganic As in macroalgae in Ireland, especially when designing feeds with high inclusion rates of macroalgae, to ensure that the established legal limits of As in the final feed are not reached. Despite the high As concentration in macroalgae, washing and soaking the biomass before cooking can reduce the amount of total As by 60% [53]. Moreover, after the digestion of washed and cooked *H. fusiforme*, only 5% of the total As content was accumulated by mice [54].

## 3. Materials and Methods

### 3.1. Macroalgal Biomass, Collection and Preparation

*L. hyperborea, L. digitata* and *A. nodosum* were harvested during the winter, summer, spring and autumn of 2016 and 2017 by Quality Sea Veg Ltd. (Co. Donegal, Ireland). Samples were cleaned from epitopes, oven-dried (50 °C, 9 days), milled and sieved to 1 mm particle size using a hammer mill (Christy and Norris, Chelmsford, UK). All the seaweed samples were then vacuum-packed and stored at room temperature for further analyses.

### 3.2. Chemical Analyses

The DM of the dried and milled macroalgae was determined by oven-drying the samples (105 °C, 16 h) and the ash contents by igniting the samples in a muffle furnace (550 °C, 6 h), following the official methods of analysis described by the AOAC.942.05 [55]. The GE of the samples was determined using an adiabatic Parr 1201 bomb calorimeter (Parr Instruments, Moline, IL, USA). The N content of the macroalgae was determined by the Dumas combustion method using a LECO FP 528 instrument (LECO Instruments UKLTD., Cheshire, UK), estimating the protein content of the samples by using the conversion factor of 4.17 as described for brown macroalgae by Biancarosa et al. [56]. The TSS were determined following the phenol-sulphuric acid assay, following the protocol described by Brummer and Cui [57]. The NDF and ADF were determined following the method described by Van Soest et al. [58] using the fibre analyser Ankom 220 (Ankom ™ Technology, Macedon, NY, USA). The EE was determined using a Soxtec apparatus (Tecator, Sweden), following the AOAC.920.39 [55]. The TG were determined enzymatically by using the kit K-YBGL (Megazyme, Bray, Ireland), following the manufacturer’s recommendations. The fucoidan content of the samples were analysed following the protocol as described by Garcia-Vaquero et al. [59] using the method modified from Usov, Smirnova and Klochkova [20]. Briefly, 1 mL of fucose standards (ranging from 0.005 to 0.1 mg/mL) and samples were added to 4.5 mL of a mixture 1:6 of water:sulfuric acid and hydrolysed for 10 min at 100 °C. The samples and standards were cooled at room temperature; 0.1 mL of 3% (*w*/*v*) cysteine hydrochloride solutions were added, and the mixtures incubated during 60 min at room temperature. The fucose content of the samples was determined against the fucose standard at effective absorbance of A396–A430 in a microplate reader (Epoch, BioTek, Winooski, VT, USA).

To determine the TPC and antioxidant activity of macroalgae, the macroalgal samples were pre-treated in 80% methanol (1:10, *w*/*v*) and placed in an orbital shaker (Heildolph instruments, Schwabach, Germany) at 170 rpm at room temperature overnight. The methanolic extracts were filtrated, evaporated, freeze-dried and stored at −20 °C before further antioxidant analyses. The TPC of macroalgae was determined using the Folin–Ciocalteu phenol reagent following the method described by Ganesan et al. [60], with slight modifications as described by Ainsworth and Gillespie [61]. Briefly, 100 μL of gallic acid standards (0.05–0.5 mg/mL) and macroalgal samples, at appropriate dilutions to fit within the calibration line, were mixed with 2 mL of a sodium carbonate solution in water (2%, *w*/*v*), followed by the addition of 100 μL 1 M Folin–Ciocalteu solution. The mixtures were incubated at room temperature for 30 min, and the absorbance of the reactions was read at 720 nm in a spectrophotometer (Epoch, BioTek, Winooski, VT, USA). The ferric reducing antioxidant power (FRAP) assay was performed following the methodology described by Bolanos de la Torre et al. [62]. Briefly, 280 µL of a FRAP working solution containing a mixture (10:1:1:1.4; *v*/*v*/*v*/*v*) of 300 mM acetate buffer, 20 mM ferric chloride, 10 mM 2,4,6-Tripyridyl-s-Triazine (TPTZ) in 40 mM HCl and Milli Q water, was added to 20 µL of macroalgal extracts (1 mg/mL) and trolox standards (15–420 µM). The mixtures were incubated (37 °C, 30 min) and the final absorbance of the reaction was read at 593 nm in a microplate reader (Epoch, BioTek, Winooski, VT, USA). The FRAP antioxidant activity of each extract is expressed as mg trolox equivalents per 100 mg of freeze-dried extract. The 2,2-diphenyl-1-picrylhydrazyl (DPPH) radical scavenging activity was performed according to Nicklisch and Waite [63] with the modifications as describe by Garcia-Vaquero et al. [64]. Briefly, macroalgal extracts and positive control (ascorbic acid) were assayed at 1 mg per mL of sample buffer (0.1 M citrate phosphate buffer with 0.3% of Triton X-100). The reaction started by adding 10 µL of 2 mM DPPH solution in methanol to each well, followed by an incubation of 30 min at room temperature in dark conditions. The percentage of DPPH inhibitory activity was calculated by subtracting the absorbance readings of the wells at 515 nm before and after the addition of the DPPH solution. All the chemical analyses were performed in triplicate.

### 3.3. Fatty Acid Profiling

The preparation of fatty acid methyl esters (FAME) for the analysis of fatty acid (FA) profiles of macroalgae were performed in a microwave system MARS 6 Express 40 (CEM Corporation, Matthews, NC, USA) following the method as described by Brunton et al. [65]. Briefly, 1.5 g of macroalgal sample, 100 μL of internal standard (IS) solution (C17:0 methyl ester at 2 mg per mL in pentane) and 10 mL of potassium hydroxide in methanol (2.5%, *w*/*v*) were saponified in microwave reaction vessels by heating the system to 130 °C for 4 min and holding the temperature for 4 min. After cooling the samples to room temperature, the methyl esterification of the fatty acids was performed by adding 15 mL of an acetyl chloride solution in methanol (5%, *v*/*v*) and heating the microwave system to 120 °C for 4 min, holding this final temperature for 2 min. After cooling the vessels to room temperature, the FAME were extracted by adding 10 mL pentane and 20 mL of saturated salt solution and shaking the mixtures. Following separation of the layers, 1.5 mL of the top pentane layer containing the FAME was aliquoted in vials containing sodium sulphate for gas chromatography (GC) analyses.

FAME were separated and quantified using a Clarus 580 Gas Chromatograph fitted with a flame ionisation detector and a capillary column CP-Sil 88 with 100 m × 0.25 mm ID in length and 0.2 µm of film thickness (Agilent, Santa Clara, California, USA). Hydrogen at a flow rate of 1.25 mL/min was used as a carrier gas using an injection volume of 0.5 µL and a split ratio of 10:1. The injector and detector temperatures were 250 °C and 270 °C, respectively. The oven temperature was set initially at 80 °C and increased to 220 °C at a rate of 6.2 °C/min, holding this temperature for 3.2 min, followed by later increases to 240 °C at 6.3 °C/min, holding this temperature for 6.5 min (runtime 35 min). The identification of fatty acids (FAs) was performed by comparison of their retention times with those of a certified reference material (SupelcoTM FAME mix; Sigma Aldrich, Arklow, Co. Wicklow, Ireland). The integration of the peaks was performed using the software TotalChrom 6.3.2 (PerkinElmer, Waltham, MA, USA), and their quantification was done on the basis of the IS. The FA content of the samples is expressed as mg per kg macroalgae on dry weight (DW) basis.

### 3.4. Essential and Toxic Metal Profiling

For the determination of essential and toxic metals, 1 g of macroalgal samples were digested with nitric acid and hydrogen peroxide in a microwave digestion system (Millestone Ethos Plus, Sorisole, Italy) by increasing the temperature of the mixtures from 25 °C to 200 °C during 10 min and maintaining the temperature for a further 10 min. After the digestion process, the samples were cooled down at room temperature and diluted to 15 mL with ultrapure water (18 MΩ cm^−1^) following the protocol previously described by López-Alonso et al. [66]. Additional processing was required for the determination of iodine (I) by treating the samples following the high temperature alkaline extraction procedure as described in EN [67].

The concentrations of essential (Ca, Co, Cr, Cu, Fe, I, Mg, Mn, Mo, Ni, P, Se and Zn) and toxic (As, Cd, Pb and Hg) metals were determined by inductively coupled plasma mass spectrometry (ICP-MS, VGElemental PlasmaQuad SOption, equipped with a micromist low-flow nebulizer) at a plasma flow rate of 14 mL/min, auxiliary gas flow rate 1 mL/min and nebulizer gas flow 0.8 mL/min, following the previously established operational conditions [66]. All the samples were analysed in triplicate, and the concentration of essential and toxic metals in the samples is expressed as mg/kg DW macroalgae.

An analytical quality control program was applied throughout the study by including blank samples and certified reference material (CRM) alongside the samples. The values of the blanks were subtracted from the sample readings, and the limits of detection of the method were calculated as 3 times the standard deviation of the reagent blanks. The limits of quantification, expressed as a concentration in the macroalgae, were calculated on the basis of the mean sample volume and total volume analysed. Analytical recoveries were determined from the CRMs SRM 1515 (Apple leaves, NIST) and BCR 279 (*Ulva Lactuca*, IRMM) with acceptable results (Supplementary Table S7 online).

### 3.5. Climatological Data

The climatological data of the region of collection of macroalgae was collected in the atmospheric research station at Mace Head by Met Éireann [68]. The temperature (°C), evaporation (mm) and solar radiation (J/cm^2^) encompassing both visible and near-visible (ultraviolet and near-infrared) were compiled monthly during the years 2016 and 2017.

### 3.6. Statistical Analyses

The influence of the macroalgae species, season and year of collection on the composition of macroalgae were analysed by multivariate general linear model in SPSS version 24.0. The differences were further analysed by either Tukey’s HSD post hoc tests or Student’s *t*-test. The variance in the data was analysed by principal component analysis (PCA) using direct Oblimin rotation with Kaiser normalisation to obtain the expected weight for each component with eigenvalues higher than 1 in SPSS version 24.0. The correlations in the data were explored using R ([69]; Version 4.0.2) with the packages “ggplot2” and “corrplot” used to generate a graphical display of the Pearson’s correlation matrix [70]. The function “cor.mtest” produced the *p*-values for each pair of input features included in the correlation matrix.

## 4. Conclusions

Overall, the composition of macroalgae was extremely variable depending on the species, season and year of collection. There was a strong negative correlation between the accumulation of protein and carbohydrates (total soluble sugars, total glucans and/or fucoidan) in the macroalgae in this study. In general, the levels of protein were high during winter and/or spring and decreased to a minimum during summer and/or autumn, while the levels of carbohydrates followed an opposite accumulation trend to that described for proteins. Positive correlations were also identified in all macroalgae between their phenolic contents and antioxidant properties (DPPH and/or FRAP). Moreover, solar radiation and evaporation seem to explain the levels of TPC and FRAP antioxidant activity in the intertidal macroalga *A. nodosum*. The levels of TPC and FRAP antioxidant activity in *A. nodosum* in the current study were in general low during the winter, increasing during spring and summer and declining during autumn. These results suggest an increased production of antioxidant compounds by the macroalgal cells in periods of increased oxidative stress damage in macroalgal species located in the upper-middle shore compared with species growing in the low-shore or sub-tidal regions.

When analysing the fatty acid profiles of macroalgae, the concentration of fatty acids was higher in *A. nodosum* compared with the other two Laminaria species. Overall, the main PUFA quantified in this study included the ω3 ALA and EPA and the ω6 LA and ARA, being the profile and relative accumulation of fatty acids in macroalgae (SFA, MUFA, ω3 and ω6) clearly differentiated between the three macroalgal species studied. The results of the current study could indicate the potential of the fatty acids profiles of macroalgae, particularly the ω3 and ω6 that cannot be synthesised by animals, to be used as biomarkers of macroalgal consumption. The mineral profiles of the three brown macroalgae of this study were rich in essential metals, particularly Ca, Mg and P. The levels of I were high in the case of the two *Laminaria* spp. The levels of toxic metals (Cd, Hg and Pb) in all the macroalgal species studied were low, while the levels of total As were relatively high and may need further research to ensure that the current legal limits of arsenic in feed are not met when designing diets containing a high percentage of macroalgae.

## Figures and Tables

**Table 1:** Proximate composition of *Laminaria digitata*, *Laminaria hyperborea* and *Ascophyllum nodosum* collected each season during the years 2016 and 2017.

Proximate Composition *	*Macroalgae* sp.	Year 2016				Year 2017			
		Winter	Spring	Summer	Autumn	Winter	Spring	Summer	Autumn
DM	*L. digitata*	91.39 ± 0.01 Bc	90.42 ± 0.01 Bd	91.88 ± 0.04 Bb	94.44 ± 0.05 Ba	93.98 ± 0.00 Ac	92.83 ± 0.04 Ad	94.13 ± 0.01 Ab	95.93 ± 0.01 Aa
Ash		34.84 ± 0.08 Aa	32.55 ± 0.05 Bc	19.92 ± 0.02 Ad	34.28 ± 0.01 Ab	32.42 ± 0.03 Bb	36.00 ± 0.03 Aa	18.96 ± 0.01 Bd	21.82 ± 0.00 Bc
GE		11.03 ± 0.03 Bc	11.13 ± 0.00 Ab	13.82 ± 0.01 Aa	11.11 ± 0.01 Bbc	11.52 ± 0.01 Ac	10.78 ± 0.03 Bd	13.78 ± 0.01 Aa	13.56 ± 0.00 Ab
EE		0.26 ± 0.05 Ab	0.51 ± 0.00 Aab	0.87 ± 0.02 Aa	0.66 ± 0.19 Aab	0.38 ± 0.03 Ac	0.03 ± 0.00 Bd	0.74 ± 0.01 Bb	1.12 ± 0.05 Aa
NDF		54.41 ± 0.40 Ab	64.01 ± 0.55 Aa	46.35 ± 0.55 Ac	55.57 ± 0.87 Ab	27.01 ± 1.37 Bc	39.23 ± 0.32 Bb	44.98 ± 0.55 Aa	42.15 ± 0.01 Bab
ADF		16.06 ± 0.02 Bc	23.79 ± 0.14 Aa	22.28 ± 0.28 Ab	15.57 ± 0.12 Bc	20.65 ± 0.26 Ab	24.69 ± 0.58 Aa	10.75 ± 0.04 Bc	23.25 ± 0.93 Aab
Protein		11.12 ± 0.76 Aa	9.15 ± 0.00 Aa	3.63 ± 0.04 Ab	5.54 ± 0.03 Ab	9.98 ± 0.09 Aa	6.92 ± 0.02 Bb	2.88 ± 0.06 Bd	4.01 ± 0.04 Bc
TSS		11.88 ± 0.13 Ac	12.41 ± 0.00 Ab	21.03 ± 0.11 Ba	10.88 ± 0.06 Bd	11.75 ± 0.07 Ac	11.19 ± 0.01 Bc	26.94 ± 0.05 Aa	20.39 ± 0.56 Ab
DM	*L. hyperborea*	90.83 ± 0.00 Bd	92.84 ± 0.03 Bc	94.28 ± 0.02 Ab	95.17 ± 0.02 Ba	95.81 ± 0.04 Aa	95.01 ± 0.00 Ab	94.19 ± 0.00 Ac	95.75 ± 0.02 Aa
Ash		30.01 ± 0.03 Ab	35.64 ± 0.04 Aa	18.36 ± 0.02 Bd	22.34 ± 0.32 Ac	30.33 ± 0.34 Ab	32.79 ± 0.04 Ba	20.82 ± 0.02 Ac	18.91 ± 0.16 Bd
GE		12.77 ± 0.04 Ac	12.00 ± 0.01 Ad	14.60 ± 0.02 Aa	13.91 ± 0.05 Bb	11.25 ± 0.11 Bc	12.28 ± 0.12 Ab	14.44 ± 0.01 Aa	14.68 ± 0.01 Aa
EE		0.76 ± 0.07 Aa	0.75 ± 0.10 Aa	0.65 ± 0.12 Aa	0.80 ± 0.01 Aa	0.60 ± 0.01 Aab	0.56 ± 0.02 Aab	0.34 ± 0.07 Ab	0.69 ± 0.06 Aa
NDF		51.56 ± 0.77 Bb	67.17 ± 0.66 Aa	40.29 ± 0.95 Ac	40.21 ± 0.05 Bc	64.17 ± 0.71 Aa	48.90 ± 0.21 Bb	44.68 ± 0.24 Ac	66.09 ± 1.20 Aa
ADF		19.81 ± 1.04 Aa	21.49 ± 0.42 Ba	22.48 ± 0.15 Aa	21.65 ± 0.08 Aa	21.30 ± 0.46 Ab	35.12 ± 0.53 Aa	20.06 ± 0.67 Ab	10.84 ± 0.00 Bc
Protein		9.98 ± 0.01 Aa	7.22 ± 0.01 Ab	2.15 ± 0.13 Ad	3.93 ± 0.06 Ac	8.02 ± 0.04 Ba	4.16 ± 0.04 Bb	2.22 ± 0.27 Ac	3.57 ± 0.00 Ab
TSS		14.49 ± 0.11 Ac	11.85 ± 0.03 Ad	19.21 ± 0.09 Bb	20.15 ± 0.21 Ba	13.73 ± 0.07 Bc	11.23 ± 0.03 Bd	20.42 ± 0.06 Ab	26.69 ± 0.05 Aa
DM	*A. nodosum*	90.38 ± 0.01 Bd	92.73 ± 0.08 Ab	91.02 ± 0.02 Bc	93.94 ± 0.04 Ba	95.30 ± 0.08 Aa	91.89 ± 0.01 Ad	92.88 ± 0.01 Ac	94.69 ± 0.00 Ab
Ash		23.31 ± 0.30 Aa	20.04 ± 0.08 Ac	17.91 ± 0.06 Bd	21.87 ± 0.09 Ab	23.98 ± 0.05 Aa	19.97 ± 0.12 Ac	19.76 ± 0.14 Ac	21.28 ± 0.22 Ab
GE		14.36 ± 0.02 Bc	15.30 ± 0.06 Ab	15.99 ± 0.05 Aa	14.56 ± 0.02 Ac	14.53 ± 0.03 Ab	14.96 ± 0.02 Aa	14.81 ± 0.04 Ba	14.44 ± 0.13 Ab
EE		3.33 ± 0.00 Ab	2.54 ± 0.04 Ac	3.82 ± 0.10 Aa	2.73 ± 0.03 Ac	3.17 ± 0.02 Aa	3.49 ± 0.28 Aa	3.50 ± 0.10 Aa	2.20 ± 0.01 Bb
NDF		53.47 ± 0.58 Aa	56.10 ± 0.55 Aa	52.67 ± 1.23 Aa	53.44 ± 0.80 Aa	49.50 ± 0.75 Ab	56.43 ± 0.36 Aa	46.64 ± 0.18 Ac	41.11 ± 0.41 Bd
ADF		21.95 ± 0.37 Ab	30.35 ± 0.34 Aa	12.15 ± 0.39 Bc	29.76 ± 0.00 Aa	16.37 ± 0.16 Bb	16.05 ± 0.56 Bb	16.26 ± 0.21 Ab	20.04 ± 0.30 Ba
Protein		6.14 ± 0.01 Aa	5.22 ± 0.09 Ab	2.68 ± 0.00 Ad	3.52 ± 0.01 Ac	4.03 ± 0.05 Bb	3.60 ± 0.02 Bc	2.25 ± 0.06 Ad	4.44 ± 0.11 Aa
TSS		13.66 ± 0.08 Ab	14.27 ± 0.07 Aa	13.34 ± 0.03 Ab	11.63 ± 0.06 Bc	12.43 ± 0.02 Bc	13.02 ± 0.15 Bb	12.33 ± 0.11 Ac	14.63 ± 0.01 Aa

* Dry matter (DM, %), ash (%), gross energy (GE, mJ/kg DW macroalgae), ether extract (EE, g/100 g DW macroalgae), neutral detergent fibre (NDF, g/100 g DW macroalgae), acid detergent fibre (ADF, g/100 g DW macroalgae), protein (g/100 g DW macroalgae) and total soluble sugars (TSS, g/100 g DW macroalgae). Results are expressed as average ± standard deviation of the mean. Different letters indicate statistical differences (*p* < 0.05) in the proximate composition of each macroalgae between different seasons within the same year (lower case letters) or differences within the same season between the years 2016 and 2017 (upper case letters).

## Data Availability

Not applicable.

## Supplementary Materials

The following are available online at https://www.mdpi.com/article/10.3390/md19040204/s1. Table S1: Fatty acid (mg/kg DW macroalgae) profile of *L. digitata* collected all seasons during the years 2016 and 2017; Table S2: Fatty acid (mg/kg DW macroalgae) profile of *L. hyperborea* collected all seasons during the years 2016 and 2017; Table S3: Fatty acid (mg/kg DW macroalgae) profile of *A. nodosum* collected all seasons during the years 2016 and 2017; Table S4: Concentration of essential and toxic trace elements (mg/kg DW macroalgae) in *L. digitata* collected all seasons during the years 2016 and 2017; Table S5: Seasonal variation of the concentration of essential and toxic trace elements (mg/kg DW macroalgae) in *L. hyperborea* collected during the years 2016 and 2017; Table S6: Seasonal variation of the concentration of essential and toxic trace elements (mg/kg DW macroalgae) in *A. nodosum* collected during the years 2016 and 2017; Table S7: Analytical quality control programme for the determination of essential and toxic metal concentrations (μg/L).
